# Qinggan Huoxue Recipe Protects against Experimental Alcoholic Liver Fibrosis through CXCL16 Inhibition

**DOI:** 10.1155/2023/5642713

**Published:** 2023-01-03

**Authors:** Ling Tang, Xiao Yu, Lu Zou, Shaobin Li, Yanqi Cheng, Yuqin Wu, Lianjun Xing, Hong Fang

**Affiliations:** ^1^Preventive Health Department of Traditional Chinese Medicine, Longhua Hospital, Shanghai University of Traditional Chinese Medicine, No. 725, Wanping South Road, Xuhui District, Shanghai 200032, China; ^2^Department II of Digestive Diseases, Longhua Hospital, Shanghai University of Traditional Chinese Medi-cine, No. 725, Wanping South Road, Xuhui District, Shanghai 200032, China; ^3^Experiment Center for Teaching & Learning, Shanghai University of Traditional Chinese Medicine, No. 1200, Cailun Road, Shanghai 201203, China

## Abstract

**Background:**

Qinggan Huoxue recipe (QGHXR), a traditional Chinese medicinal formula, has a protective effect against liver fibrosis. However, the underlying mechanisms remain unclear.

**Objective:**

This study investigated the antifibrotic role of QGHXR and its underlying mechanisms.

**Methods:**

The composition of QGHXR was determined using ultra performance liquid chromatography-tandem mass spectrometry (UPLC-MS/MS). Female C57BL/6J mice were fed either a Lieber–DeCarli liquid diet or pair-fed control diet and intraperitoneally injected with CCl_4_ for 8 weeks (*n* = 8). In week 5, the mice were administered 100, 200, and 400 mg/kg QGHXR via oral gavage daily for 4 weeks.

**Results:**

UPLC-MS result showed that QGHXR contained 45 compounds including salvianolic acid A, scutellarin, baicalin, rutin, and chai saponin D. QGHXR alleviated pathological alterations in the liver. The alanine aminotransferase (ALT) level was reduced to 44.88 ± 4.39 U/L, aspartate aminotransferase (AST) to 76.25 ± 4.17 U/L, alkaline phosphatase (ALP) to 60.75 ± 5.41 U/L, and acetaldehyde to 38.54 ± 1.01 U/L compared with that of the control group (ALT 72.38 ± 5.19 U/L, AST 119.63 ± 9.82 U/L, and ALP 98.63 ± 6.71 U/L and acetaldehyde 64.86 ± 4.70 U/L). QGHXR inhibited lipid overproduction and fibrotic gene expression. The serum concentration of chemokine C-X-C ligand 16 (CXCL16) was reduced to 62.83 ± 6.80 pg/ml compared with that of the control group (130.91 ± 13.72 pg/mL). QGHXR downregulated CXCL16 mRNA and protein expressions. Pharmacological CXCL16 treatment reversed the QGHXR-induced protective effects in ethanol plus CCl_4_ fed mice. QGHXR reduced CXCL16 levels (91.97 ± 5.86 pg/ml) in LPS-stimulated RAW264.7 cells compared with that of the control group (148.68 ± 8.62 pg/ml) and inhibited toll-like receptor 4 and nuclear factor-kappa B phosphorylation.

**Conclusions:**

This study demonstrated that QGHXR mitigates experimental alcoholic liver fibrosis by CXCL16 inhibition, and may be considered a potential therapeutic agent for treating liver fibrosis.

## 1. Introduction

Liver fibrosis is a common pathological consequence of chronic liver diseases, including hepatitis B and C, alcohol abuse, and nonalcoholic steatohepatitis. It is characterized by excessive intercellular accumulation of extracellular matrix (ECM) in the liver [1]. Advanced liver fibrosis may progress to cirrhosis, which is responsible for liver failure and death [2]. High mortality associated with alcoholic liver fibrosis is caused by upper gastrointestinal bleeding, infection, and hepatic encephalopathy, which are attributed to alcohol-induced liver cirrhosis. Although research has been ongoing for many years to identify novel therapeutic targets for alcoholic liver disease (ALD), no medication has been approved by the FDA for the treatment of ALD [3]. Currently, the only effective treatment for liver fibrosis is liver transplantation [4]. Therefore, the development of novel antifibrotic agents for hepatic fibrosis is urgently required.

Alcoholic liver fibrosis is characterized by dyslipidemia, inflammation, and fibrotic deposition. Inhibition of inflammatory mediators, including chemokines, has been shown to ameliorate alcoholic liver fibrosis [5]. Chemokines, a family of small cytokines that can induce leukocyte migration, have been identified as central regulators of liver fibrosis [6]. Chemokine C-X-C ligand 16 (CXCL16), which has soluble and transmembrane forms, is a ligand for the CXC chemokine receptor 6 (CXCR6) [7]. Several studies have emphasized the crucial role of CXCL16 in the progression of liver fibrosis [8–10]. CXCL16 is highly expressed in patients with hepatic fibrosis [9]. In addition, substantial antifibrotic efficacy has been observed in therapies that deactivate CXCL16 signaling [11]. Herein, we hypothesized that CXCL16 might be a critical target for alcoholic liver fibrosis.

As complementary and alternative medicine, traditional Chinese medicine (TCM) is receiving increasing attention worldwide for the prevention and treatment of various diseases [12, 13]. TCM exhibits hepatoprotective effects against alcoholic liver fibrosis via heat-clearing and detoxifying effects, which are critical for antipyretic and anti-inflammatory action, and dissipation of blood stasis in the theory of TCM [14]. Qinggan Huoxue recipe (QGHXR), which is composed of *Radix bupleuri*, *Radix scutellariae*, *Radix salviae miltiorrhizae*, *Carapax trionycis*, and *Radix puerariae*, is a traditional Chinese medicine that possesses prominent biological activities, including anti-inflammatory, antiapoptotic, antisteatosis, and antifibrotic [15, 16]. The previous study showed that QGHXR prevents alcoholic liver fibrosis in rats by decreasing hepatocyte apoptosis and inhibiting epithelial-to-mesenchymal transition [15]. In addition, QGHXR showed beneficial effects and increased survival rates in rats with acute liver failure [17]. However, the molecular mechanism of the protective effect of QGHXR against alcoholic liver fibrosis remains elusive. Although CXCL16 exists as a crucial role in the progression of liver fibrosis, the role of CXCL16 in QGHXR-mediated antifibrotic effects has not yet been elucidated. Therefore, in this study, we treated mice with QGHXR to determine whether QGHXR could protect against ethanol + CCl_4_-induced liver fibrosis in a CXCL16-dependent manner.

## 2. Materials and Methods

### 2.1. Reagents and Drugs

CCl_4_ was purchased from Sigma-Aldrich (St. Louis, MO, USA). CXCL16 was purchased from PeproTech (Rocky Hill, NJ, USA). QGHXR is composed of *Radix bupleuri* 9 g, *Radix scutellariae* 9 g, *Radix salviae miltiorrhizae* 15 g, *Carapax trionycis* 9 g, and *Radix puerariae* 15 g. All herbs were obtained from Shanghai Sunbow Co. Ltd. (Shanghai, China) and authenticated by Associate Professor Yan Xi (Department of Pharmacy of Longhua Hospital). The herbs were processed at the Department of Pharmacy of Longhua Hospital (Shanghai, China) to produce QGHXR.

### 2.2. Animals and Treatments

Female C57BL/6J mice were obtained from Shanghai Sipper-BK Laboratory Animal Co., Ltd. (Shanghai, China). Mice were housed in a specific pathogen-free environment at 23 ± 1°C with a 12 h light/dark cycle. All procedures were conducted according to protocols approved by the local ethics committee for animal research studies at the Shanghai University of Traditional Chinese Medicine. Female C57BL/6J mice were either fed a Lieber–DeCarli liquid diet containing 4% ethanol or pair-fed a control diet, and mice were intraperitoneally injected with a 2.5% solution of CCl_4_ in olive oil (0.1 mL/kg) for 8 weeks, as previously described [18]. In week 5, the mice were administered 100, 200, and 400 mg/kg QGHXR via oral gavage daily for 4 weeks. Mice were divided into five groups (8 mice per group, 23–25 g): pair-fed group (negative control), ethanol + CCl_4_ group (control group), ethanol + CCl_4_ + 100, ethanol + CCl_4_ + 200, and ethanol + CCl_4_ + 400 mg/kg QGHXR group. For CXCL16 treatment, in week 5, mice were administered with 100, 200, and 400 mg/kg QGHXR via oral gavage daily and 0.1 mg/kg CXCL16 (i.v., once per week) for 4 weeks. Mice were divided into four groups (8 mice per group, 23–25 g): ethanol + CCl_4_ group (control group), ethanol + CCl_4_ + 400 mg/kg QGHXR group, ethanol + CCl_4_ + CXCL16 group, and ethanol + CCl_4_ + CXCL16 + 400 mg/kg QGHXR group. CCl_4_ was dissolved in olive oil (1 : 40, 2.5%) as a stock solution. The mice were sacrificed, and the livers were collected for analysis.

### 2.3. Qualitative Analysis Using Ultra Performance Liquid Chromatography-Tandem Mass Spectrometry (UPLC-MS/MS)

**Figure 1:**
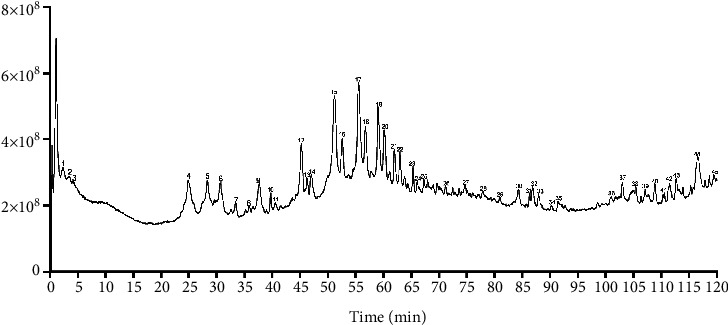
UPLC-MS chromatogram for QGHXR. 1: threonine; 2: arginine; 3: histidine; 4: salvianolic acid A; 5: scutellarin; 6: baicalin; 7: puerarin apigenin; 8: puerarin-6″-O-xyloside; 9: 3′-hydroxypuerarin apigenin; 10: puerarin A; 11: puerarin B; 12: daidzein; 13: isotanshinone IIB; 14: tanshinone II; 15: rutin; 16: *β*-3,4-dihydroxyphenyllactic acid; 17: chai saponin D; 18: wogonin; 19: salvianolic acid B; 20: hydroxytuberosone; 21: saikosaponin C; 22: purpuric acid; 23: salvianolic acid C; 24: indirubin; 25: salvigenin; 26: daidzein-4′-glucoside; 27: danshensu; 28: kaempferol; 29: quercetin; 30: viscidulin I; 31: daucosterol; 32: trichopterin A; 33: isorhamnetin; 34: rosmarinic acid; 35: baicalein I; 36: baicalein II; 37: daidzein; 38: chrysin; 39: carthamine; 40: wogonin; 41: *β*-sitosterol; 42: genistein; 43: formononetin; 44: 3′-hydroxypuerarin; 45: baicalein II.

UPLC-MS analysis was performed using a Shimadzu LCMS-8050 mass spectrometer coupled with a UPLC chromatograph. Chromatographic separation was achieved using a Shim-pack XR-ODS C18 column (2.0 × 75 mm, 1.6 *μ*m). The parameters used are as follows: capillary voltage, 15 kV; spray flow, 3 L/min; heating gas flow, 10 L/min; interface temperature, 350°C; DL temperature, 250°C; heating block temperature, 400°C; and drying airflow rate, 10 L/min. Gradient elution was performed using acetonitrile and water (2%–98%) for 120 min. The MS spectra of the compounds were imported into the natural compound database for comparison, and the results with a similarity of more than 90% were taken for LCMS comparison analysis and qualitative analysis with the corresponding standards. The LC-MS/MS chromatograms of the 45 compounds in QGHXR are shown in Figure 1.

### 2.4. Serum Biochemical Assays

Serum levels of aspartate aminotransferase (AST), alanine aminotransferase (ALT), alkaline phosphatase (ALP), and CXCL16 were determined using biochemical kits (Nanjing Jiancheng Bioengineering Institute, Nanjing, China) by following the manufacturer's instructions.

### 2.5. Determination of Hepatic Lipid Levels

Hepatic lipid levels were assessed according to the manufacturer's instructions of the relevant kits (Nanjing Jiancheng Bioengineering Institute).

### 2.6. Real-Time PCR

Total RNA was extracted from the liver tissues using TRIzol (Invitrogen, Carlsbad, CA, USA) and reverse-transcribed into cDNA using the PrimeScript RT kit (Takara). Real-time PCR was performed using the two-step quantitative RT-PCR method (Bio-Rad, Hercules, CA, USA). The target gene expression was normalized to the average expression of the housekeeping gene glyceraldehyde-3-phosphate dehydrogenase (GAPDH). The primers used are listed in Table 1.

### 2.7. Western Blot Assay

To extract protein, the whole liver tissue was homogenized in ice-cold lysis buffer, as previously described [19]. Protein samples were subjected to SDS-PAGE and electroblotted to PVDF membranes (Bio-Rad). After blocking with 5% bovine serum albumin for 1 h, the membranes were incubated with primary antibody against CXCL16 or CXCR6 at 4°C overnight. The membranes were washed in TBST (0.1% Tween) and incubated with the appropriate HRP-conjugated secondary antibody for 90 min at room temperature. The signals were visualized using chemiluminescence and exposure to X-ray films, and the relative expression level of the target protein was normalized to that of GAPDH.

### 2.8. Oil Red O (ORO) and Masson Staining

ORO staining was performed to evaluate hepatic steatosis. The ORO working solution was prepared by diluting the ORO stock solution (5 g/L in isopropanol) with distilled H_2_O at a ratio of 3 : 2. Liver sections were fixed in 60% isopropanol and stained with ORO working solution for 15 min. The stained lipid droplets were observed under a light microscope.

Fibrosis was assessed using Masson's trichrome staining kit (Nanjing Jiancheng Bioengineering Institute, Nanjing, China), following the manufacturer's protocol. The sections were then visualized under a light microscope.

### 2.9. Hematoxylin and Eosin (H&E) Staining

An H&E staining kit (Beyotime) was used for standard histological assessment. Samples were fixed in 4% paraformaldehyde, embedded in paraffin, and cut into 5 *μ*m sections. The sections were then stained with H&E and observed under a light microscope.

### 2.10. Cell Culture

The murine macrophage cell line RAW264.7 was purchased from Shanghai Institute of Cell Biology (Shanghai, China) and maintained in Dulbecco's modified Eagle's medium (DMEM) supplemented with 100 U/mL penicillin, 100 *μ*g/mL streptomycin, and 10% fetal bovine serum in a 5% carbon dioxide atmosphere incubator at 37°C.

### 2.11. Statistical Analysis

Data were analyzed using one-way analysis of variance (ANOVA) with SPSS (version 17.0; IBM, Chicago, IL, USA). Dunnett's multiple comparison *post hoc* test was used to determine group differences. Data are expressed as mean ± SEM. Statistical significance was set at *p* < 0.05.

## 3. Results

### 3.1. QGHXR Rescues Mice from Ethanol plus CCl_4_-Induced Liver Injury

**Figure 2:**
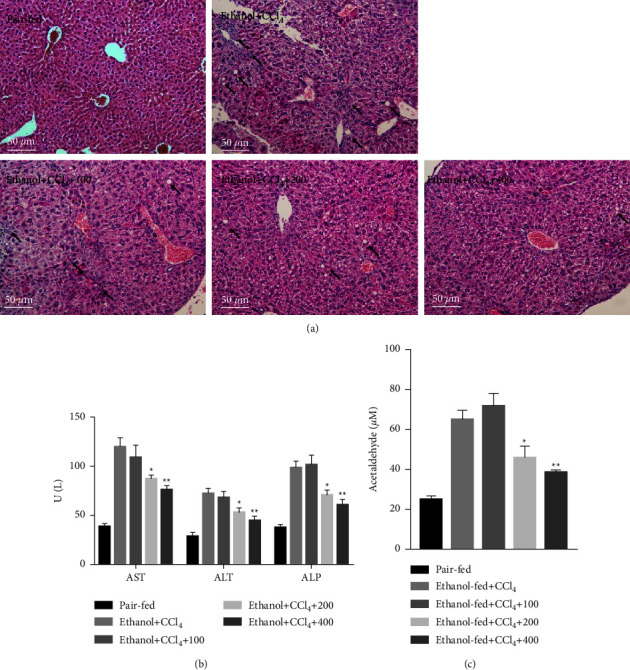
QGHXR attenuates liver injury induced by CCl_4_ in chronic ethanol-fed mice. C57BL/6J mice were either fed a Lieber–DeCarli liquid diet containing 4% ethanol or pair-fed a control diet, and mice were intraperitoneally injected with CCl_4_ (0.1 mL/kg) for 8 weeks. At week 5, mice were administered with 100, 200, and 400 mg/kg QGHXR by gavage daily for 4 weeks. (a) Representative hematoxylin and eosin staining, magnification 100x. (b) The levels of serum AST, ALT, and ALP. (c) Hepatic acetaldehyde levels. The values represent means ± SEM (*n* = 8). ^*∗*^*p* <  0.05, ^*∗∗*^*p* <  0.01 vs. ethanol + CCl_4_.

First, the composition of QGHXR was determined using UPLC-MS. The chromatogram showed that QGHXR contained 45 compounds (Figure 1). To determine the effect of QGHXR on ethanol plus CCl_4_-induced liver injury, histological examination was conducted using H&E staining. The levels of AST, ALT, ATP, and acetaldehyde were measured using the relevant biochemical kits. As indicated in Figure 2(a), treatment with QGHXR reduced ethanol plus CCl_4_-induced hepatocyte vacuolar degeneration and inflammatory cell infiltration (the arrows indicated the regions). QGHXR also improved several biochemical markers of liver injury, including serum AST, ALT, and ALP, and promoted hepatic acetaldehyde clearance (Figures 2(b) and 2(c)). Taken together, these results indicate that QGHXR administration attenuates ethanol plus CCl_4_-induced liver injury.

### 3.2. QGHXR Reduces Ethanol plus CCl_4_-Induced Hepatic Steatosis

**Figure 3:**
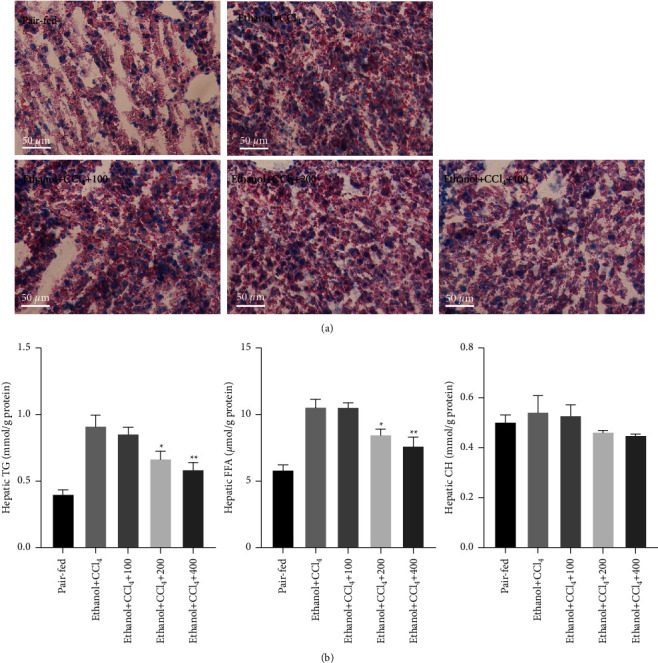
QGHXR inhibits hepatic steatosis in ethanol plus CCl_4_-administrated mice. C57BL/6J mice were either fed a Lieber–DeCarli liquid diet containing 4% ethanol or pair-fed a control diet, and mice were intraperitoneally injected with CCl_4_ (0.1 mL/kg) for 8 weeks. At week 5, mice were administered with 100, 200, and 400 mg/kg QGHXR by gavage daily for 4 weeks. (a) Representative oil red O staining, 100x. (b) Hepatic TG, FFA, and CH levels. The values represent means ± SEM (*n* = 8). ^*∗*^*p* < 0.05, ^*∗∗*^*p* < 0.01 vs. ethanol + CCl_4_.

To examine whether QGHXR alleviates hepatic steatosis *in vivo*, hepatic lipid droplets and levels of triglycerides (TGs), free fatty acids (FFAs), and total cholesterol (CH) were evaluated in mice (Figure 3). ORO staining showed more lipid droplets in ethanol plus CCl_4_-treated mice than in the pair-fed group, which were reduced with QGHXR treatment. Biochemical lipid measurements demonstrated severe TG and FFA deposition in the livers of ethanol + CCl_4_ model mice relative to pair-fed animals, which was markedly attenuated by QGHXR administration. However, no significant difference was detected in the hepatic CH levels. Collectively, these data suggest that QGHXR protects mice from ethanol plus CCl_4_-induced hepatic steatosis.

### 3.3. QGHXR Alleviates Ethanol plus CCl_4_-Induced Liver Fibrosis

**Figure 4:**
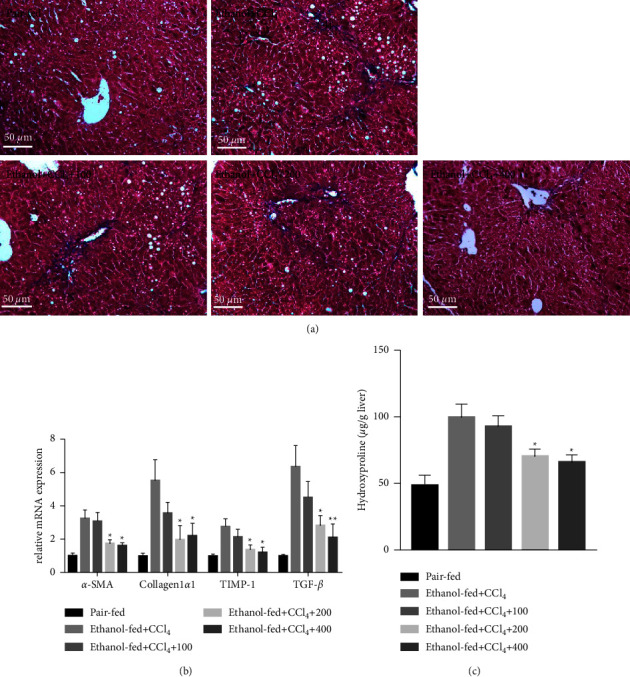
QGHXR ameliorates liver fibrosis after ethanol plus CCl_4_ administration in mice. C57BL/6J mice were either fed a Lieber–DeCarli liquid diet containing 4% ethanol or pair-fed a control diet, and mice were intraperitoneally injected with CCl_4_ (0.1 mL/kg) for 8 weeks. At week 5, mice were administered with 100, 200, and 400 mg/kg QGHXR by gavage daily for 4 weeks. (a) Representative Masson staining, 100x. (b) The fibrotic gene expression in the liver. (c) Hepatic hydroxyproline levels. The values represent means ± SEM (*n* = 8). ^*∗*^*p* < 0.05, ^*∗∗*^*p* < 0.01 vs. ethanol + CCl_4_.

Then, we analyzed the effects of QGHXR intervention on ethanol plus CCl_4_-induced liver fibrosis. Trichrome-stained liver sections from ethanol plus CCl_4_ model mice demonstrated remarkable fibrosis (Figure 4(a)). Furthermore, the expression of fibrotic genes, including *α*-SMA, Collagen1*α*1, TIMP-1, and TGF-*β* was substantially upregulated in ethanol-fed mice after CCl_4_ injection (Figure 4(b)). Similarly, hepatic levels of hydroxyproline (a marker of collagen content) were higher in the ethanol + CCl_4_ group than in pair-fed mice (Figure 4(c)). However, QGHXR treatment decreased trichrome-stained areas of the liver tissue compared to the untreated ethanol + CCl_4_ model mice. Furthermore, *α*-SMA, Collagen1*α*1, TIMP-1, and TGF-*β* gene expression and hydroxyproline content were substantially reduced by QGHXR treatment.

### 3.4. QGHXR Decreases CXCL16 Expression in Ethanol plus CCl_4_-Induced Liver Fibrosis

**Figure 5:**
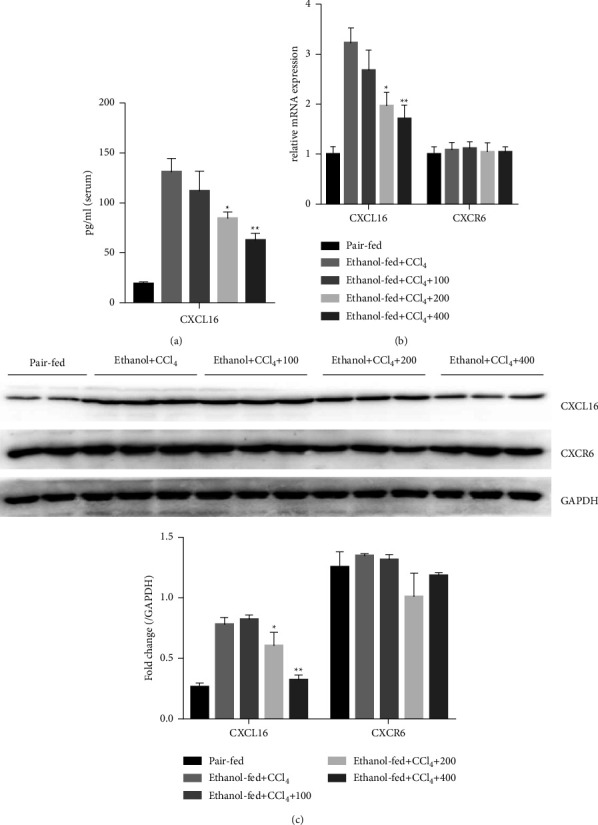
QGHXR reduced CXCL16 levels after ethanol plus CCl_4_ administration in mice. C57BL/6J mice were either fed a Lieber–DeCarli liquid diet containing 4% ethanol or pair-fed a control diet, and mice were intraperitoneally injected with CCl_4_ (0.1 mL/kg) for 8 weeks. At week 5, mice were administered with 100, 200, and 400 mg/kg QGHXR by gavage daily for 4 weeks. (a) The level of CXCL16 in serum were determined by an ELISA assay. (b) The levels of CXCL16 and CXCR6 mRNA in the liver were determined by real-time PCR. (c) The levels of CXCL16 and CXCR6 protein expression in the liver were determined by western blot. The arbitrary units were analyzed by ImageJ software. The values represent means ± SEM (*n* = 8). ^*∗*^*p* < 0.05, ^*∗∗*^*p* < 0.01 vs. ethanol + CCl_4_.

To explore the mechanisms underlying the beneficial effects of QGHXR on liver fibrosis, we studied the CXCL16/CXCR6 axis in ethanol plus CCl_4_-treated mice following QGHXR administration. Ethanol plus CCl_4_ treatment resulted in an increase in serum CXCL16 concentration (Figure 5(a)). Consistent with the ELISA data, the mRNA and protein levels of CXCL16 were elevated in the livers of ethanol plus CCl_4_-treated animals (Figures 5(b) and 5(c)). Despite the increase in CXCL16 expression, no change was observed in the CXCR6 levels. It is noteworthy that QGHXR administration profoundly reversed serum CXCL16 levels, and similar reductions were observed in the liver CXCL16 mRNA and protein levels. Taken together, these results suggest that CXCL16 signaling is involved in the QGHXR-induced protective effects in experimental alcoholic liver fibrosis.

### 3.5. CXCL16 Treatment Reversed QGHXR-Induced Protective Effects in Ethanol plus CCl_4_-Treated Mice

**Figure 6:**
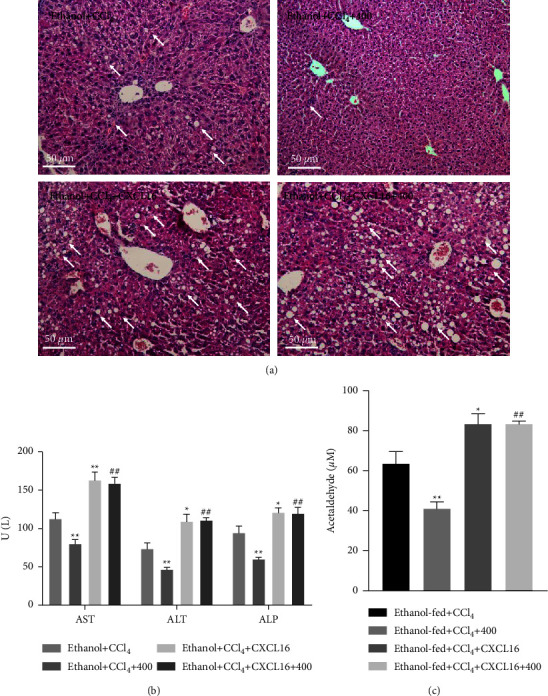
CXCL16 treatment abolishes QGHXR-generated protective effects on liver injury in ethanol-fed mice with CCl_4_ injection. C57BL/6J mice were fed a Lieber–DeCarli liquid diet containing 4% ethanol and intraperitoneally injected with CCl_4_ (0.1 mL/kg) for 8 weeks. At week 5, mice were administered with 400 mg/kg QGHXR by gavage daily and 0.1 mg/kg CXCL16 (i.v., once per week) for 4 weeks. (a) Representative hematoxylin and eosin staining, magnification 100×. (b) The levels of serum AST, ALT, and ALP. (c) Hepatic acetaldehyde levels. The values represent means ± SEM (*n* = 8). ^*∗∗*^*p* < 0.01 vs. ethanol + CCl4; ^##^*p* <  0.01 vs. ethanol + CCl_4_ + 400.

**Figure 7:**
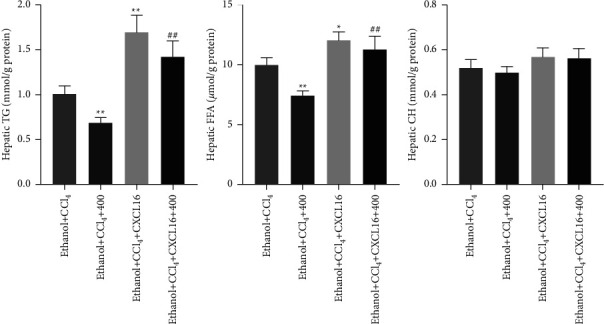
CXCL16 treatment compromised QGHXR-generated protective effects on hepatic steatosis in ethanol-fed mice with CCl_4_ injection. C57BL/6J mice were fed a Lieber–DeCarli liquid diet containing 4% ethanol and intraperitoneally injected with CCl_4_ (0.1 mL/kg) for 8 weeks. At week 5, mice were administered with 400 mg/kg QGHXR by gavage daily and 0.1 mg/kg CXCL16 (i.v., once per week) for 4 weeks. Hepatic TG, FFA, and CH levels were assayed using relevant commercial kits. The values represent means ± SEM (*n* = 8). ^*∗∗*^*p* < 0.01 vs. ethanol + CCl4; ^##^*p* < 0.01 vs. ethanol + CCl_4_ + 400.

**Figure 8:**
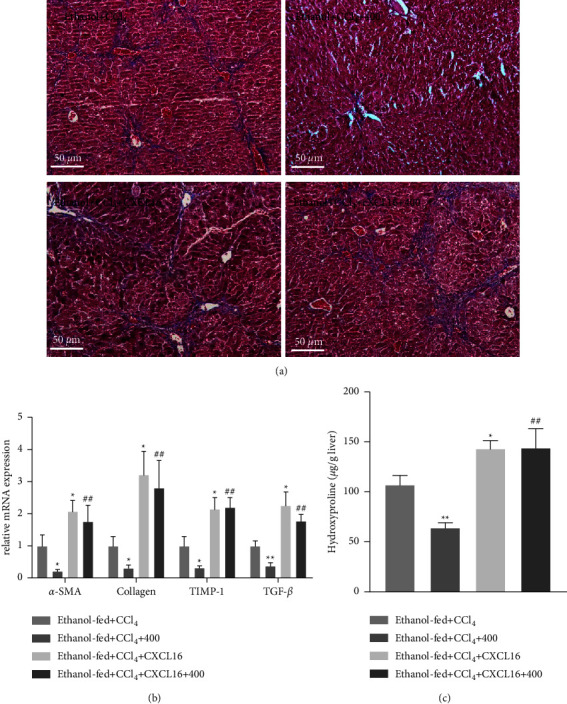
CXCL16 treatment compromised QGHXR-generated protective effects on liver fibrosis in ethanol-fed mice with CCl_4_ injection. C57BL/6J mice were fed a Lieber–DeCarli liquid diet containing 4% ethanol and intraperitoneally injected with CCl_4_ (0.1 mL/kg) for 8 weeks. At week 5, mice were administered with 400 mg/kg QGHXR by gavage daily and 0.1 mg/kg CXCL16 (i.v., once per week) for 4 weeks. (a) Representative Masson staining, 100×. (b) The fibrotic gene expression in the liver. (c) Hepatic hydroxyproline levels. The values represent means ± SEM (*n* = 8). ^*∗*^*p* < 0.05, ^*∗∗*^*p* < 0.01 vs. ethanol + CCl_4_; ^##^*p* < 0.01 vs. ethanol + CCl_4_ + 400.

To confirm the role of CXCL16 in QGHXR-induced protective effects in experimental liver fibrosis, pharmacological CXCL16 treatment was used in this study. As shown in Figure 6, CXCL16 treatment abolished QGHXR-induced attenuation of liver injury, as evidenced by increased liver injury in H&E-stained liver sections and elevated serum AST, ALT, and ALP production and hepatic acetaldehyde in the ethanol + CCl_4_ + CXCL16 + 400 group when compared with those of the ethanol  + CCl_4_ + 400 group. In addition, hepatic TG and FFA levels were markedly depleted after QGHXR treatment alone but increased by CXCL16 and QGHXR cotreatment, suggesting that CXCL16 treatment suppressed QGHXR-induced effects on hepatic steatosis (Figure 7). No difference was found in the hepatic CH levels. Then, we focused on the influence of CXCL16 treatment on the QGHXR-induced protective effects in liver fibrosis. As shown in Figure 8, collagen accumulation, fibrotic gene expression (*α*-SMA, Collagen1*α*1, TIMP-1, and TGF-*β*), and hydroxyproline levels were inhibited by QGHXR administration and reversed by CXCL16 treatment. Based on the abovementioned findings, CXCL16 inhibition is required in QGHXR-exerted antifibrotic effects.

### 3.6. QGHXR Inhibits Toll-like Receptor 4 (TLR4) and Phosphorylated Nuclear Factor-Kappa B (p-NF-*κ*B) Expression *In Vitro* and *In Vivo*

**Figure 9:**
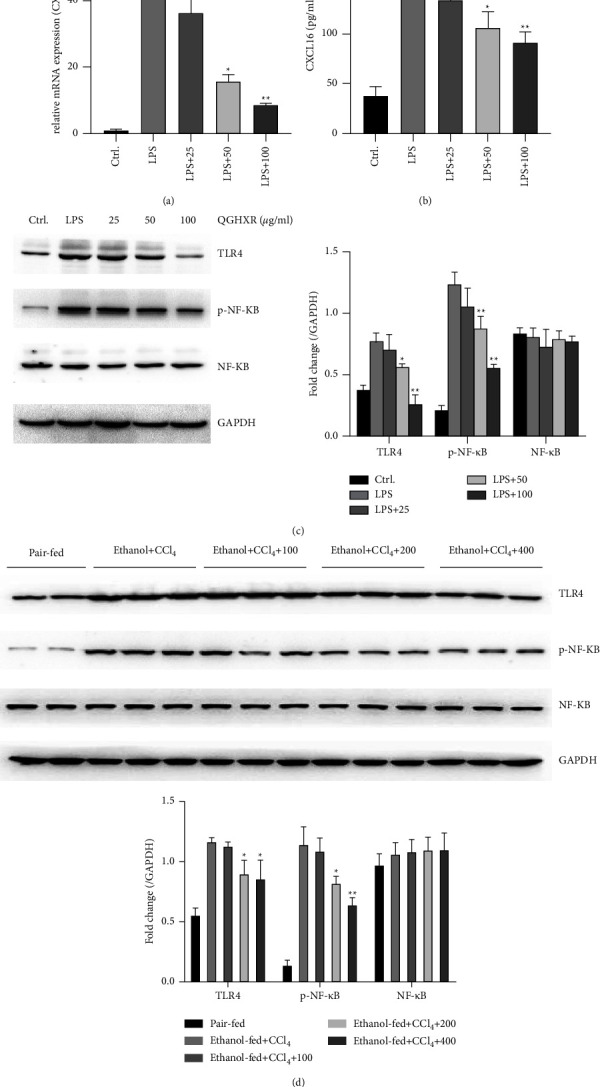
QGHXR inhibits TLR4 and p-NF-*κ*B expression *in vitro* and *in vivo*. (a) The cells stimulated by 100 ng/ml LPS were treated with different concentrations of QGHXR for 24 h. The mRNA level of CXCL16 was determined by real-time PCR. (b) The level of CXCL16 in the cell culture supernatant was determined by ELISA assay. (c) The levels of TLR4, p-NF-*κ*B, and NF-*κ*B protein expression in cells were determined by western blot. (d) The levels of TLR4, p-NF-*κ*B, and NF-*κ*B protein expression in the ethanol plus CCl_4_-administrated mice liver were determined by western blot. The arbitrary units were analyzed by ImageJ software. The values represent means ± SEM. ^*∗*^*p* < 0.05, ^*∗∗*^*p* < 0.01 vs. control.

According to a previous study [10], CXCL16 is strongly expressed in macrophages in the murine liver, whereas natural killer T (NKT) and CD4/8 T cells express CXCR6. To investigate the mechanism underlying CXCL16 inhibition, RAW264 a murine macrophage cell line (RAW264.7) was treated with lipopolysaccharide (LPS), which activates the TLR4/NF-*κ*B pathway to induce CXCL16 expression. As shown in Figures 9(a) and 9(b), QGHXR treatment inhibited LPS-induced CXCL16 mRNA expression and protein levels in a dose-dependent manner. Western blot assay shows that QGHXR also inhibited TLR4 and p-NF-*κ*B levels in RAW264.7 cells (Figure 9(c)). To confirm the effect of QGHXR on the TLR4/NF-*κ*B pathway, TLR4 and p-NF-*κ*B expression levels were determined in the mouse liver. In line with the *in vitro* results, QGHXR also remarkably inhibited TLR4 and p-NF-*κ*B expression in the ethanol plus CCl_4_-administrated mouse liver. These results indicate that the TLR4/NF-*κ*B pathway is responsible for the inhibition of CXCL16 by QGHXR.

## 4. Discussion

Qinggan Huoxue recipe (QGHXR) is a Chinese herbal prescription that exerts beneficial effects on liver fibrosis. In this study, we explored the antifibrotic effects of QGHXR. We found that QGHXR alleviated liver injury, hepatic steatosis, and hepatic fibrosis and downregulated CXCL16 expression in ethanol plus CCl_4_-induced liver fibrosis. Furthermore, pharmacological CXCL16 administration ablated the QGHXR-induced protective effects in experimental alcoholic liver fibrosis.

Ethanol feeding and CCl_4_injection-induced murine models have been widely used to study liver fibrosis [18, 20]. CCl_4_ is a hepatotoxic chemical that effectively induces liver fibrosis in rodents, and ethanol exposure was shown to accelerate CCl_4_-induced liver fibrosis [21, 22]. Liver damage, including hepatocyte necrosis, steatosis, and fibrosis, has been reported in ethanol plus CCl_4_-treated animals [20, 23]. In this study, mice that underwent ethanol feeding and CCl_4_ injection displayed obvious histopathological changes through H&E staining and QGHXR treatment reduced hepatocyte vacuolar degeneration and inflammatory cell infiltration. UPLC-MS/MS result showed that QGHXR contained 45 compounds including salvianolic acid A, scutellarin, baicalin, rutin, and chai saponin D. The effects of these natural products in QGHXR on liver disease were extensively reported. Salvianolic acid A suppressed CCl4-induced liver fibrosis in mice by inhibiting inflammation and oxidative stress [24]. Scutellarin protected against CCl_4_-liver injury in mice by repressing CYP2E1 and I*κ*B*α*/NF-*κ*B signaling pathways and modulating the gut microbiota [25]. Baicalin ameliorates alcohol-induced hepatic steatosis by suppressing SREBP1c-elicited PNPLA3 competitive binding to ATGL [26]. With a multi-ingredient and multitarget-pathway pharmacological action, QGHXR as a traditional Chinese medicine is compatible with the complex pathogenesis of ALD to mitigate it. It was believed that the integrated regulation of QGHXR makes it more competitive than any other chemical drugs or active ingredients.

Chemokines are involved in the development of steatosis, inflammation, and fibrosis in alcoholic liver disease [27]. Thus, it has been hypothesized that inhibition of chemokines is the key mechanism of QGHXR-modulated alcoholic liver fibrosis. CXCL16, a small-inducible cytokine B16 and SR-PSOX, belongs to the type I membrane proteins of the CXC chemokine family. CXCL16 is a multifunctional chemokine that combines the scavenger receptor function with the properties of inflammatory chemokines [28]. CXCL16 is universally believed to serve a pivotal molecule that controls inflammatory and immune reactions. CXCL16 reduces local inflammation and drives chemokine-dependent activation of leukocytes in the liver. CXCR6, the receptor of CXCL16, is implicated in multiple inflammatory dysfunctions. Previous studies suggested that CXCL16 plays a vital role in liver disorders. CXCL16 exists on the surface of antigen-presenting cells and hepatocytes in patients with hepatic disorders [29]. Hepatic CXCL16 mRNA and protein levels are increased in gallstones accompanied by liver injury [30]. The administration of an anti-CXCL16 antibody increased the survival of mice with immunological liver injury by reducing the infiltration of T lymphocytes into the liver tissue [31]. Activation of CXCL16 signaling enhances lipid deposition in fatty livers of apolipoprotein E knockout mice [32]. The serum concentration of CXCL16 is positively correlated with the activities of ALT and AST in patients with gallstones [7]. More severe hepatic pathological alterations have been observed in CXCL16-deficient mice [29]. The above evidence indicates that CXCL16 may play a critical role controlling liver repair in QGHXR-treated mice. Our results confirmed that QGHXR administration attenuated histopathological changes, inhibited transaminase activity, and suppressed dyslipidemia.

CXCL16 also plays a key role in liver fibrosis. Increased hepatic CXCL16 mRNA expression was found in patients with liver fibrosis compared to controls [10]. NKT cells drive hematopoietic stem cell activation and liver fibrosis by stimulating CXCL16 in chronic liver injury [10]. CXCL16 and CXCR6 have been proposed to be responsible for anchoring activated NKT cells [10]. Metformin, a first-line agent for type 2 diabetes treatment, was demonstrated to reduce the gene expression levels of several fibrotic markers, including *α*-SMA, Collagen1*α*1, and TGF-*β*1, which is associated with the reduction of CXCL16 mRNA expression [11]. The current study showed that ethanol feeding and CCl_4_ injection resulted in increased serum CXCL16 concentrations and upregulated hepatic CXCL16 mRNA and protein expression, suggesting the involvement of CXCL16 in the development of liver fibrosis. Importantly, it has been reported that in the murine liver, CXCL16 is strongly expressed by the endothelium and macrophages, whereas lymphocytes, including NKT, NK, CD4, and CD8 T cells, express CXCR6 [10]. In the present study, we found that QGHXR did not alter CXCR6 levels, indicating that lymphocytes may not be the target cells of QGHXR. Moreover, excessive alcohol intake impairs the intestinal barrier and increases intestinal permeability, resulting in endotoxins entering the portal venous circulation, which in turn induces proinflammatory cytokine and chemokine production in Kupffer cells [33]. QGHXR inhibited endotoxin-induced CXCL16 levels and blocked the TLR4/NF-*κ*B pathway in macrophages (Figure 9). Consistent with the *in vitro* results, QGHXR intervention robustly decreased CXCL16 levels in the serum and liver of ethanol-fed mice injected with CCl_4_, indicating that the reduced CXCL16 level is partially attributed to QGHXR-induced protective effects in experimental liver fibrosis. Moreover, pharmacological CXCL16 treatment abolished QGHXR-induced beneficial effects in the ethanol plus CCl_4_ mouse model, as evidenced by liver function biomarkers, dyslipidemia, and fibrotic indices. These results further demonstrate that CXCL16 deactivation is required for the antifibrotic efficacy of QGHXR.

## 5. Conclusions

This study demonstrates that QGHXR protects mice from ethanol plus CCl_4_-induced liver fibrosis. The protective properties of QGHXR are dependent on CXCL16 deactivation. Our results reveal the potential mechanism underlying QGHXR-induced antifibrotic effects and suggest that CXCL16 could serve as a novel target for the treatment of liver fibrosis.

## Figures and Tables

**Table 1:** List of primers used in the study.

Gene	Forward primer (5′–3′)	Reverse primer (5′–3′)
CXCL16	GTTGCAGTCCAAAAGCGTG	GTCTGGGTACTGGCTTGAG
CXCR6	AGCACACTTCACTCTGGAAC	TTGAAGAGCCAGAAATCTCCC
TIMP1	CTCAAAGACCTATAGTGCTGGC	CAAAGTGACGGCTCTGGTAG
*α*-SMA	GTGAAGAGGAAGACAGCACAG	GCCCATTCCAACCATTACTCC
Collagen1*α*1	CATAAAGGGTCATCGTGGCT	TTGAGTCCGTCTTTGCCAG
TGF-*β*	CCTGAGTGGCTGTCTTTTGA	CGTGGAGTTTGTTATCTTTGCTG

## Data Availability

The data used to support the findings of this study are available from the corresponding authors upon request.
